# Optimization of a Binary Concrete Crack Self-Healing System Containing Bacteria and Oxygen

**DOI:** 10.3390/ma10020116

**Published:** 2017-01-26

**Authors:** Jinlong Zhang, Bixia Mai, Tingwei Cai, Jiayi Luo, Wanhan Wu, Bing Liu, Ningxu Han, Feng Xing, Xu Deng

**Affiliations:** 1 Shenzhen Key Laboratory of Marine Bioresource and Eco-environmental Science, College of Life Sciences and Oceanography, Shenzhen University, Shenzhen 518060, China; zjlwood@szu.edu.cn (J.Z.); 871830180max@gmail.com (B.M.); 2015300071@email.szu.edu.cn (T.C.); 2015300183@email.szu.edu.cn (J.L.); 201430004@email.szu.edu.cn (W.W.); 2 Guangdong Province Key Laboratory of Durability for Marine Civil Engineering, The Key Laboratory on Durability of Civil Engineering in Shenzhen, School of Civil Engineering, Shenzhen University, Shenzhen 518060, China; liubing0708@szu.edu.cn (B.L.); nxhan@szu.edu.cn (N.H.); 3 Key Laboratory of Earthquake Engineering and Engineering Vibration, Institute of Engineering Mechanics, China Earthquake Administration, Harbin 150086, China

**Keywords:** microbially induced calcium precipitation, spore vialility, oxygen supply, self healing, concrete crack, *Bacillus* sp.

## Abstract

An optimized strategy for the enhancement of microbially induced calcium precipitation including spore viability ensurance, nutrient selection and O_2_ supply was developed. Firstly, an optimal yeast extract concentration of 5 g/L in sporulation medium was determined based on viable spore yield and spore viability. Furthermore, the effects of certain influential factors on microbial calcium precipitation process of H4 in the presence of oxygen releasing tablet (ORT) were evaluated. The results showed that CaO_2_ is preferable to other peroxides in improving the calcium precipitation by H4. H4 strain is able to precipitate a highly insoluble calcium at the CaO_2_ dosage range of 7.5–12.5 g/L, and the most suitable spore concentration is 6 × 10^8^ spores/ml when the spore viability (viable spore ratio) is approximately 50%. Lactate is the best carbon source and nitrate is the best nitrogen source for aerobic incubation. This work has laid a foundation of ternary self-healing system containing bacteria, ORT, and nutrients, which will be promising for the self-healing of cracks deep inside the concrete structure.

## 1. Introduction

Recently, there has been growing interest in microbial self-healing process due to its potential in longlasting, efficient and environment-friendly crack repair of concrete [1,2]. Self-healing of concrete cracks by bacteria is based on microbial-induced calcium carbonate (CaCO_3_) precipitation (MICP) [3,4,5]. There are also many examples of MICP in nature, including calcite formation in soils, limestone caves, seas, and soda lakes [6]. Based on the biosynthetic pathway of carbonate, the mechanisms of MICP that have been applied in the self-healing concrete can be classified into several types, such as ureolysis, aerobic respiration, and anaerobic respiration [7]. Four different factors regulate MICP performance, which include: (i) concentration of soluble calcium, (ii) concentration of carbonate, (iii) pH, and (iv) availability of nucleation sites for the formation of calcium carbonate crystal [8]. 

The central issue in the microbial self-healing process is the calcium precipitating activity of bacteria [3,7]. Several strategies that have been applied by different investigators for enhancement of the calcium precipitation are : (i) Bacterial species screening. Jan Dick et al. investigated the ureolytic calcium precipitation process of *B. pasteurii*, *B. cereus* and *B. sphaericus* and found *B. sphaericus* strains are most suitable for coherent calcite production on degraded limestone [9]. Zhang et al. obtained a high-efficiency calcium precipitating bacterial strain designated as H4 through a bacterial isolation and screening procedure based on the decrease of soluble calcium [3]. (ii) Bacterial protection by using carriers. Light weight aggregates [10,11,12,13], diatomaceous earth [14], silica gel [15], polyurethane [15] and hydrogel-based microcapsule [16] were used as bacterial protective vehicle in different researches to improve bacterial survival rate and crack repair efficiency. (iii) Maintenance of aerobic condition through utilizing oxygen releasing compound(ORC). Zhang et al. tested the effect of CaO_2_ on the bacterial induced calcium precipitation and found that the O_2_ released by CaO_2_ dramatically increased the quantity of calcite [7]. 

However, few previous studies took the spore viability into account. George et al. investigated the amount of CaCO_3_ precipitated by bacteria under different cell concentration and found the optimal bacterial cell concentration is 2.3 × 10^8^ cells/ml [17]. Zhang et al. investigated the influence of spore concentration on calcium precipitation and the result showed that a spore concentration of more than 4 × 10^7^ spore/ml is necessary for effective calcium precipitation [3]. The cell/spore concentration plays a key role in the bacterial induced calcium precipitation. However, not all spores can germinate. Some superdormant spores or dead spores are not able to ‘wake up’ and grow to vegetative cells [18,19], thus consequently unable to induce calcium precipitation. Given that bacteria is incorporated into the concrete in the form of dormant spore [3,10], how to ensure the viability of spore is also important for the performance of bacterial self-healing concrete. Therefore, it is necessary to establish a procedure to improve the production of high-viability spores.

Moreover, a binary self-healing system containing CaO_2_ (a kind of ORC) and spore has been developed in our previous study [7]. ORC is selected and utilized to enhance the calcium precipitation deep inside the concrete crack where O_2_ is not available. In the presence of oxygen, spores are proved to germinate more effectively and maintain high metabolic activity, resulting in 3 times induced calcium precipitation more than that obtained without oxygen supply [7]. However, considering that the presence of ORC leads to alteration of environmental situations like dissolved oxygen (DO) and pH, different influential factors on the improvement of microbial calcium precipitation in the presence of ORC need to be further optimized, such as spore concentration, CaO_2_ dosage, carbon source and nitrogen source. 

In the present study, we developed an integrated strategy for the enhancement of MICP including spore viability ensurance, nutrient selection and O_2_ supply. Firstly, the influence of yeast extract concentration in spore-producing medium on spore viability and calcium precipitation was evaluated. Furthermore, suitable peroxide was screened based on the bacterial calcium precipitation detection. Besides, the effects of such influential factors as carbon source, nitrogen source, spore concentration and ORC dosage on the MICP in the presence of ORC were investigated. This work aimed to improve microbial calcium precipitation behavior in the presence of oxygen and further lay a foundation for the application of ternary self-healing system containing bacteria, ORC, and nutrients in our future study.

## 2. Materials and Methods

### 2.1. Strain

A bacterial strain H4, identified to be a type of *Bacillus* species with high calcium precipitation activity, was isolated from the sediment samples collected from a mangrove conservation area in Shenzhen Bay. The strain is deposited in the China General Microbiological Culture Collection Center (CGMCC) with a deposit number of 9629 [3].

### 2.2. Spore Preparation

A modified sporulating medium (MSP medium) based on that in Jonkers’ study [10] was used as basic medium for spore production of strain H4, which contained (1 L): NH_4_NO_3_ 0.3 g, KH_2_PO_4_ 0.02 g, CaCl_2_·2H_2_O 0.225 g, KCl 0.476 g, MgCl_2_·6H_2_O 0.2 g, MnSO_4_·2H_2_O 0.01 g, yeast extract 3 g, soluble starch 1 g, NaHCO_3_ 4.2 g and Na_2_CO_3_ 5.3 g. The pH of the medium is about 9.7. Strain H4 was grown overnight at 30 °C in alkaline LB broth containing NaHCO_3_ (4.2 g/L) and Na_2_CO_3_ (5.3 g/L), and then 8 ml of grown culture was inoculated into 100 mL fresh MSP medium. The cultivation was carried out for 4–5 days in a series of 500 mL Erlenmeyer flasks with 100 mL working volume in a rotary incubator shaker at 30 °C, 150 rpm. To obtain spore powder, spore suspension was freeze-dried and stored in a vacuum drier at 20 °C for further use. Per gram of dry spore powder contained about 1 × 10^12^ spores.

### 2.3. Preparation of Oxygen-Releasing Tablets

Oxygen releasing tablets (ORTs) were prepared by compressing peroxide and lactic acid together. For CaO_2_-ORT, powders of calcium peroxide, lactic acid and spore were crushed, ground, sieved by a 150 μm sieve (100 mesh), and then mixed at a ratio of 9:1:0.1 (*w*/*w*). The resulting powder mixture was compressed into tablets using a single punch tablet machine with 3-mm (diameter) flat surface punches. Each tablet contains 25 mg powder mixture. In one tablet, the amount of peroxide CaO_2_, lactic acid and spore powder was 22.28, 2.47 and 0.25 mg, respectively. The ZnO_2_-ORT and MgO_2_-ORT were prepared exactly in the same way except the replacement of CaO_2_ by ZnO_2_ or MgO_2_. 

### 2.4. Calcium Precipitation Determination

The calcium precipitation was determined by the tube method as described in the previous study [7]. The basic medium was CPM medium. CPM contained (g/L): L-sodium lactate 7.5, NaNO_3_ 2, MgCl_2_ 0.09, KH_2_PO_4_ 0.01, CaCl_2_ 1.67, inosine 0.67, NaCl 23.9, Na_2_SO_4_ 4, KCl 0.68, KBr 0.098, H_3_BO_3_ 0.026, NaF 0.0029, SrCl_2_ 0.0014, *N*-Cyclohexyl-2-aminoethanesulfonic acid (CHES) 20.7. The CHES was adjusted to approximately 9.5 with 6 M NaOH. ORT was added to provide a stable oxygen release. For all samples, 200 μL paraffin oil was added into the tube to form an oil layer on the surface of the solution as a barrier against O_2_ diffusion [20]. The cultures were incubated at 30 °C for 30 days. pH, soluble Ca^2+^ concentration, and the amount of precipitates were measured at 30 days. The soluble Ca^2+^ concentration in the solution was detected by OCPC method as described previously [3,7]. To measure the amount of precipitates, glass tubes were washed with distilled water at least three times to remove the cultures and calcium hydroxide, and then 2 mL of 1 M HCl was added into each tube to dissolve the wall-adhered precipitates to form CaCl_2_. The concentration of consulting free Ca^2+^ was determined using the same method mentioned above. The amount of calcium precipitates in each tube was expressed as insoluble Ca^2+^ (mM). Results are expressed as the mean value ± the standard deviation.

### 2.5. Optimization of Spore Production Process

The effect of yeast extract in MSP medium on the spore yield, spore viability and bacterial calcium precipitation was evaluated. The spores obtained from MSP with different concentrations of yeast extract were respectively used for calcium precipitation experiment. The yeast extract was prepared at different concentrations (0, 0.5, 1, 2, 3, 4, 5, 6, 7 g/L). Spore yield (*SY*) was determined with a direct counting chamber under a phase contrast microscope. Viable spore yield (*VSY*) was determined by bacterial colony counting method after a heat treatment (60 °C, 30 min) to the spore samples. All experiments had at least 3 replicates and the results are expressed as the mean value ± the standard deviation. Spore viability (*SV*) was calculated as follows:

*SV* = *VSY*/*SY* × 100% (1)

### 2.6. Optimization of the Bacterial Calcium Precipitation Process

Calcium precipitation performances with different ORT types (CaO_2_-ORT, ZnO_2_-ORT and MgO_2_-ORT) were investigated to select a suitable oxygen releasing compound and the control is the incubation system without addition of peroxide. Considering CaO_2_ releases soluble Ca^2+^ in the calcium precipitation process, the powder of CaSO_4_ with equivalent amount of calcium to that in oxygen-releasing tablets was also compressed separately and used as extra calcium sources in calcium precipitation experiment using ZnO_2_-ORT and MgO_2_-ORT. Glucose, sucrose, soluble starch, sodium formate, sodium glutamate and sodium acetate were respectively prepared at 5 g/L for replacement of the sodium lactate in CPM to optimize the carbon source and the CPM medium without carbon source was used as control. For nitrogen source, ammonium chloride, ammonium nitrate, urea, sodium glutamate, beef extract, yeast extract, tryptone and bacteriological peptone were respectively prepared at 2 g/L to replace the sodium nitrate and the CPM medium without carbon source was used as control. After the best carbon source and nitrogen source were determined, the effects of carbon source concentration and nitrogen source concentration, spore concentration and CaO_2_ dosage were further evaluated. Incubation was performed at 30 °C for 30 days. All experiments had at least 3 replicates and the results are expressed as the mean value ± the standard deviation.

## 3. Results

### 3.1. Effect of Yeast Extract on Spore Production and Calcium Precipitation

**Figure 1:**
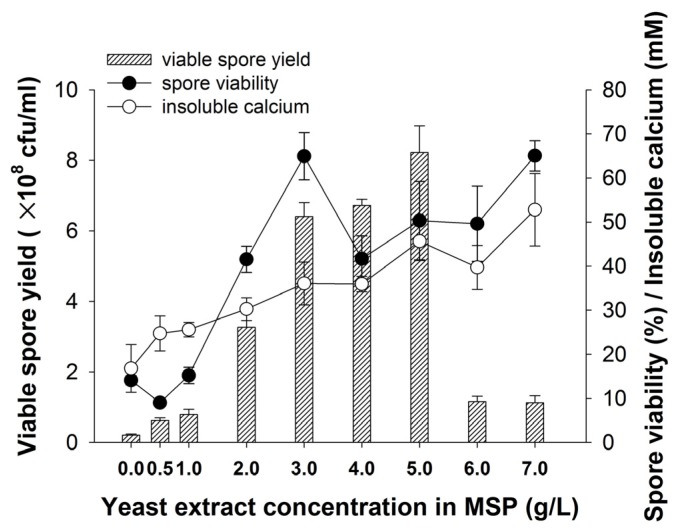
Effect of yeast extract on viable spore yield, spore viability and calcium precipitation.

In order to optimize bacterial calcium precipitation process, we focused on the effect of yeast extract in sporulation medium MSP on spore production and calcium precipitation induced by the spore in calcium precipitation medium CPM. The results are given in Figure 1. It is evident that yeast extract in MSP is highly important for the improvement of both spore production in MSP and calcium precipitation in CPM. Remarkable increase of the spore viability, viable spore yield and calcium precipitation can be observed with the increase of yeast extract concentration from 0 to 5 g/L in MSP. Compared to MSP medium without yeast extract, the spore viability, viable spore yield and calcium precipitation in CPM are respectively improved 3.5, 4 and 3 times at the yeast extract concentration of 5 g/L. However, yeast extract concentrations higher than 5 g/L in MSP result in decline of viable spore yield from 8.2 × 10^8^ to only about 1 × 10^8^ cfu/mL, because most of the vegetative cells did not sporulate (data not shown). Therefore, 5 g/L is the optimal yeast extract concentration in MSP for both spore production and calcium precipitation induced by spores in CPM. Besides, there is a significant positive correlation between spore viability and calcium precipitation as yeast extract concentration in MSP is from 4.0 to 7.0 g/L, possibly indicating that spore viability, as one of the common parameters to evaluate spore quality, poses an important impact on the bacterial calcium precipitation. 

### 3.2. Effect of Different Peroxides and CaO_2_ Dosage on Calcium Precipitation

**Figure 2:**
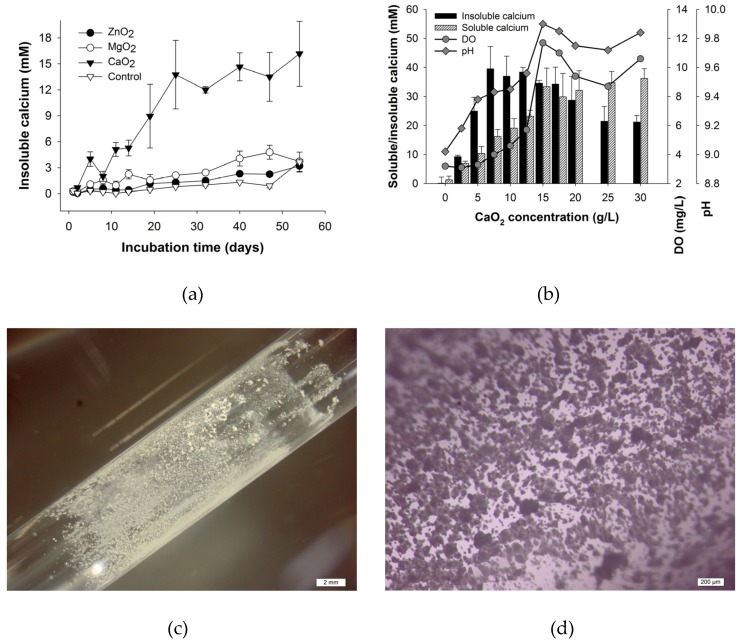

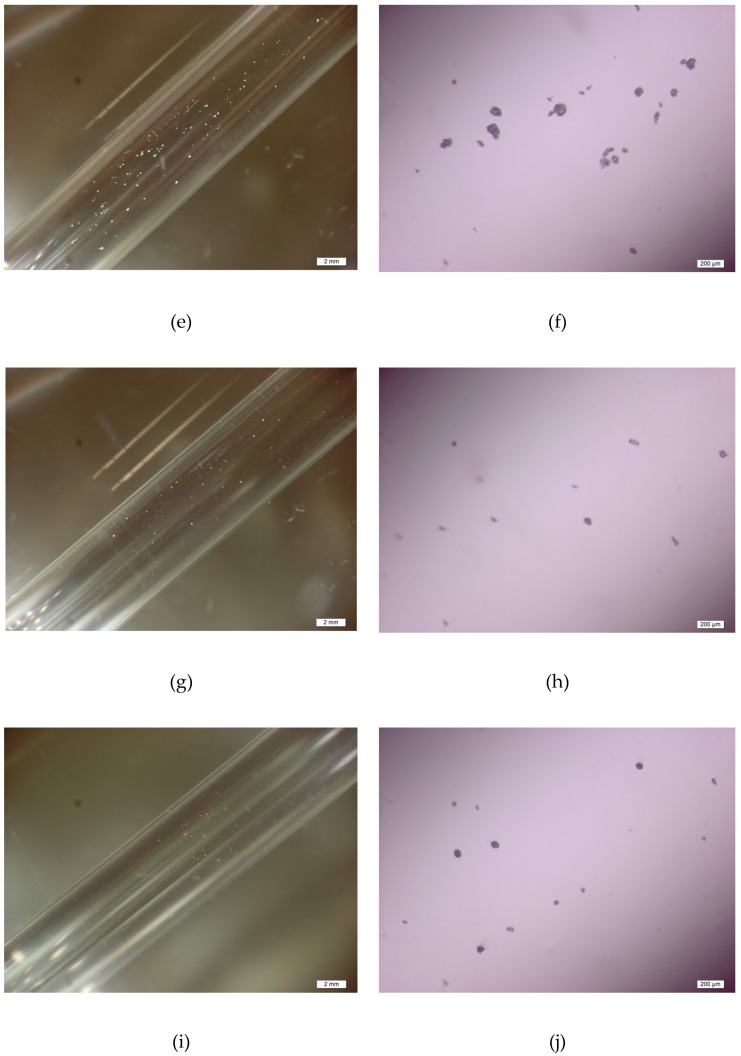
Effect of different peroxides (**a**) and CaO_2_ dosage (**b**) on calcium precipitation and stereomicroscope micrographs of the crystals in calcium precipitation experiments using CaO_2_-ORT (**c,d**), MgO_2_-ORT (**e,f**), ZnO_2_-ORT (**g,h**) and control (**i,j**). The scales are 2 mm (**c,e,g,i**) and 200 μm (**d,f,h,j**), respectively.

As mentioned above, CaO_2_ resulted in a much more increase of DO with comparison to ZnO_2_ and MgO_2_. In this work, the bacterial induced calcium precipitation was furtherly investigated with addition of these 3 peroxides respectively. As shown in Figure 2a, only little calcium precipitation was observed for all the 3 peroxides at the first 8 days. After 8 days, CaCO_3_ started to form only in the tubes where CaO_2_ was added. At the beginning stage of calcium precipitation, no notable difference in the formation of CaCO_3_ was shown among the tubes with different peroxides. However, for the tubes with addition of CaO_2_, the amount of insoluble Ca^2+^ increased sharply from 8 days. It is evident that CaO_2_ can dramatically increase the calcium precipitation while ZnO_2_ and MgO_2_ failed. As shown in the stereomicroscope micrographs (Figure 2c,d), a large amount of crystals grew on the wall of the glass tube with CaO_2_.

In CPM medium, CaO_2_ reacts with water and forms O_2_ and Ca(OH)_2_. O_2_ supply is beneficial for the calcium precipitation. However, Ca(OH)_2_ might inhibit bacterial calcium precipitation because of the high alkalinity and high calcium concentration [7]. In order to select a suitable CaO_2_ dosage, the effect of different CaO_2_ dosage on calcium precipitation was investigated (Figure 2b). The insoluble calcium precipitated by H4 is less than 1 mM with no CaO_2_ provided. However, a sharp increase of calcium precipitation occurs as CaO_2_ dosage increases from 0 to 7.5 g/L. Further increase in the CaO_2_ dosage from 10 to 30 g/L results in a decline of insoluble calcium concentration to approximately 20 mM. Therefore, the most suitable CaO_2_ dosage is 7.5 g/L. Moreover, with the increase of CaO_2_ dosage, soluble calcium concentration, pH and DO firstly increase and then keep relatively stable. 

### 3.3. Effect of Spore Concentration on the Calcium Precipitation

**Figure 3:**
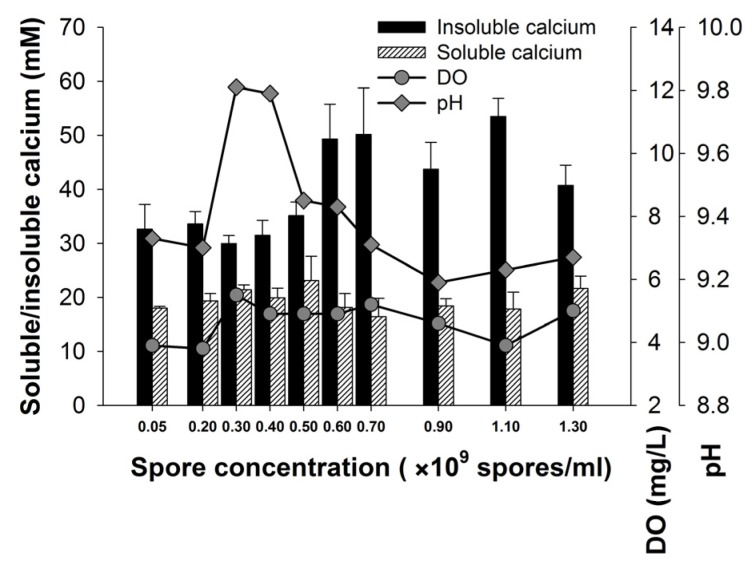
Effect of spore dosage on the calcium precipitation.

The bacterial induced calcium precipitation depends on the production of CO_3_^2−^ via bacterial metabolism and the availability of nucleation sites (macromolecules on the cell surface). From this point of view, an adequate amount of spores should be necessary to ensure high efficiency of calcium precipitation. Our experimental result in Figure 3 shows that if the spore concentration is lower than 5 × 10^8^ spores/mL, the insoluble calcium induced by H4 is less than 35 mM. However, an increase of the spore concentration from 5 × 10^8^ to 7 × 10^8^ spores/mL results in a significant increase of insoluble calcium up to 50.2 mM. A spore concentration of 1.1 × 10^9^ spores/mL could precipitate a further increase of insoluble calcium to 53.5 mM, but this increase appears to be economically ineffective, considering the cost of the production of bacterial spores. Therefore, 6 × 10^8^ spores/mL is a suitable spore concentration (SV is approximately 50%) for H4 to achieve a high level of calcium precipitation.

### 3.4. Effect of Carbon and Nitrogen Sources on the Calcium Precipitation

**Figure 4:**
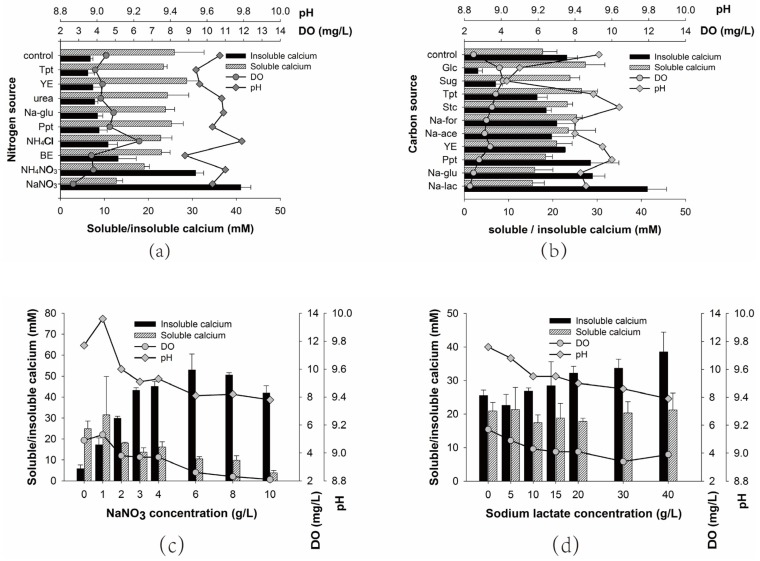
The effect of carbon source, nitrogen source and their concentrations (**c**,**d**) on bacteria induced calcium precipitation. (**a**) Na-glu sodium glutamate, YE yeast extract, BE beef extract, Tpt tryptone, Ppt microbiological peptone; (**b**) Na-for sodium formate, Sug sugar, Glc glucose, Stc starch, Na-ace sodium acetate, Na-lac sodium lactate, YE yeast extract, Tpt tryptone, Na-glu sodium glutamate, Ppt microbiological peptone.

As illustrated in Figure 4a,b, lactate and nitrate confer the highest insoluble calcium on H4. The insoluble calcium induced by H4 with tryptone, yeast extract, urea, Na-glutamate, peptone, NH_4_Cl or beef extract as nitrogen source is only about 10 mM, which is similar to that of the control with no nitrogen added. On the other hand, carbon sources like glucose and sugar exhibit the least insoluble calcium, even less than that of the control with no extra carbon source added. However, one oxygen-releasing tablet contains 2 mg of lactic acid as pH adjusting agent, which can be used as carbon source. The inhibition of glucose and sugar on calcium precipitation might be due to the acid production metabolism of H4, since pH drops to 9.0 when glucose or sugar is used as carbon source. 

Calcium precipitation under different concentration of carbon and nitrogen sources is shown in Figure 4c,d. It is clear that the presence of a nitrogen source is highly important for calcium precipitation. The insoluble calcium induced by H4 is only 5.8 mM with no nitrate provided. However, if the initial concentration of sodium nitrate is 3 g/L, a remarkable increase of insoluble calcium occurs, going up to 43 mM. Further increase in the nitrate concentration from 3 g/L only results in a gentle increase of insoluble calcium to approximately 50 mM (Figure 4c). On the other hand, an increase in the lactate concentration from 0 to 40 g/L gradually enhanced the calcium precipitation (Figure 4d). Surprisingly, H4 showed an insoluble calcium of 25 mM in the absence of lactate, which might be due to the fact that the lactic acid in the ORTs can be utilized as carbon source. Furthermore, the increase of nitrate from 0 to 3 g/L results in an insoluble calcium rise of 37 mM, while the increase of lactate from 0 to 40 g/L merely leads to a 13 mM increase of insoluble calcium, implying that the nitrogen source might be more important than the nitrogen source in terms of calcium precipitation. Besides, DO, pH and soluble calcium are found to negatively correlate with calcium precipitation. The low DO or pH might result from high respiratory and metabolic activities of H4.

## 4. Discussion

### 4.1. The Effect of Yeast Extract on Spore Production and Calcium Precipitation

The production of spores is the first step for the preparation of bacterial self-healing concrete. A high spore viability is the foundation for the improvement of self-healing efficiency. The cultivation conditions that induce sporulation, such as temperature, pH, nutrient and ion content, largely influence the features of the formed spores, including productivity and viability. Our results show that more viable spores can be obtained by increasing the concentration of yeast extract in the medium. Guizelini et al. carried out a sequence of a three-step optimization of variables and found yeast extract exerted statistically significant positive effects on sporulation, in terms of spore productivity [21]. According to Rao‘s research [22], vegetative cells initiate sporulation only after cell density reaches about 10^8^ cells/ml, even under ideal conditions. Yeast extract is a nutrient-rich medium component that facilitates bacterial cells to grow to a high cell density. Besides, the presence of yeast extract in MSP medium also improved spore viability. This suggests that certain components of the yeast extract can affect spore viability. However, what kind of components of yeast extract can affect spores viability is still not clear. Penna et al. found that increasing concentrations of yeast extract in the sporulation medium enhanced heat resistance of spores [23]. This founding is consistent with our results, because the determination of both viable spore yield and spore viability in our experiment is based on enumerating colony forming bacteria through plate counting after a heat treatment (60 °C, 30 min) to the spore samples. The low spore viability at lower concentrations of yeast extract is presumably due to the lack of essential nutrilites in the medium. The spores that are harvested from the medium with higher concentrations of yeast extract result in more calcium precipitation. At a certain amount of spores, the calcium precipitation primarily depends on the production of CO_3_^2−^ via bacterial metabolism [3]. A high spore viability ensures a sufficient amount of viable germinating or germinated cells for high efficiency of CO_3_^2−^ production.

### 4.2. Effect of Different Peroxides and CaO_2_ Dosage on Calcium Precipitation

Considering that bacterial self-healing of concrete cracks normally takes 2–3 weeks or even longer [10,24], a lasting and stable oxygen supply might be able to enhance the calcium precipitation, particularly in the cracks deep inside the concrete where oxygen is generally lack [7,13]. Our previous study proved the supply of oxygen can be realized by peroxides [7]. However, suitable peroxide should be selected in terms of the behavior of bacterial calcium precipitation. Among the 3 tested peroxides, CaO_2_ significantly increased calcium precipitation in at least 50 days as compared with other peroxides and control, suggesting a high-efficient and stable calcium precipitation. Conversely, ZnO_2_ and MgO_2_ only resulted in a slight increase of calcium precipitation. This might be because O_2_ release of ZnO_2_ is suppressed in alkaline situation. A visible oxygen release was reported when ZnO_2_ was used at moderate pH [25]. Finally, another advantage of CaO_2_ is that not only O_2_ but also Ca^2+^ are produced from the reaction of CaO_2_ with water, which may be served as the replenishment of Ca^2+^. The minerals are likely calcium carbonate-based and form due to bacterial metabolic conversion of sodium lactate according to the following reaction:

C_3_H_5_O_3_Na + 6CaO_2_ + H_2_O → 3CaCO_3_ + 3Ca(OH)_2_ + NaOH (2)

It can also be noted from Figure 2b that high CaO_2_ dosages slightly suppress the bacterial calcium precipitation. When the CaO_2_ dosage is more than 12.5 g/L, the calcium precipitation by H4 is inhibited, even though DO is higher than that at lower CaO_2_ dosage. However, given the fact that pH and soluble calcium concentration maintain high levels with CaO_2_ dosage more than 12.5 g/L, the inhibition might be due to the high alkalinity and the presence of too much soluble calcium. In our previous research, the most suitable pH range and soluble calcium concentration for calcium precipitation by H4 were found to be 10.0–10.5 and 30 mM, respectively [3]. In self-healing concrete, the oxygen release of peroxides is triggered by the crack formation and water ingress through the concrete crack. The oxygen release is slow since CaO_2_ is slightly soluble.

### 4.3. The Effect of Carbon and Nitrogen Sources on Calcium Precipitation

Carbon and nitrogen sources are important influential factors for cell growth and metabolism. As reported, based on the measurement of calcium precipitating activity in 96-well plate without addition of CaO_2_, carbon sources such as lactate and acetate, and nitrogen sources such as nitrate and urea are preferable to other carbon/nitrogen sources in terms of calcium precipitation induction of H4 [3]. Since the addition of CaO_2_ leads to alteration in the soluble calcium concentration, pH and DO [7], the nutrition requirements of H4 for calcium precipitation might be different. Therefore, it is necessary to investigate the effect of nutrient types and concentration on calcium precipitation in the presence of CaO_2_. Our experimental results reveal that sodium lactate and sodium nitrate are the best carbon and nitrogen sources for H4 in the presence of ORT. This finding is consistent with our previous research, although great differences exist in the calcium precipitation determining methods and the cultivation conditions. In previous study H4 grew in the absence of oxygen, whereas in the current research H4 was cultivated in 2 mL glass tubes with ORT added. As exhibited above, the addition of ORT changes pH, DO and soluble calcium of the medium, and all of those are important factors for calcium precipitation. Even under such different conditions, lactate and nitrate have been proven to be the best nutrient components for H4 to induce calcium precipitation. 

Incorporation of lactate into concrete has been found to result in a slight increase in the compressive strength of concrete [10], suggesting that lactate is a suitable carbon source for bacterial self-healing of concrete cracks. Tziviloglou et al. used calcium lactate as carbon source and yeast extract as nitrogen source to improve the liquid tightness recovery of concrete cracks and found that water tightness increased significantly [11]. Similarly, Wiktor and Jonkers used similar carbon and nitrogen sources to heal concrete cracks and their experimental results showed a crack-healing up to 0.46 mm wide [12]. Besides, since nitrate does not reduce the concrete strength, it is commonly used as a type of early strength agent of concrete [26]. Ersan et al. also stated that the addition of nitrate did not have a major impact on both setting times and compressive strengths of mortar specimens [27]. Therefore, it can be concluded that lactate and nitrate are suitable carbon and nitrogen sources for H4 in the self-healing process.

## 5. Conclusions

The viable spore yield and spore viability at different yeast extract concentrations were measured and compared. The calcium precipitation by H4 under different conditions (spores viability, spore concentration, peroxide types, CaO_2_ dosage, carbon/nitrogen source and their suitable concentration) were evaluated. The following conclusions are drawn:

1. The optimal yeast extract concentration for both spore production and calcium precipitation is 5 g/L.
2. CaO_2_ is the best oxygen-releasing compound that can improve the bacterial calcium precipitation and the most suitable CaO_2_ dosage is 7.5 g/L.
3. The suitable spore concentration for H4 to achieve a high level of calcium precipitation is 6×10^8^ spores/mL when the spore viability is approximately 50%.
4. Lactate is the best carbon source and nitrate is the best nitrogen source for H4 in the self-healing process of concrete crack with/without oxygen supply
